# Systemic effects of oxytocin on male sexual activity *via* the spinal ejaculation generator in rats

**DOI:** 10.1080/19420889.2021.1902056

**Published:** 2021-03-29

**Authors:** Takumi Oti, Tatsuya Sakamoto, Hirotaka Sakamoto

**Affiliations:** a Ushimado Marine Institute (UMI), Graduate School of Natural Science and Technology, Okayama University, Setouchi, Japan; b Department of Biological Sciences, Faculty of Science, Kanagawa University, Hiratsuka, Japan

**Keywords:** Spinal cord, oxytocin, gastrin-releasing peptide, male sexual activity, spinal ejaculation generator, hypothalamus, systemic treatment, rat

## Abstract

Oxytocin is produced in the hypothalamus and stimulates uterine contraction and milk ejection. While many people consider oxytocin to be a female hormone, it is reported that, in men, the plasma oxytocin level increases markedly after ejaculation. However, this aspect of oxytocin physiology is poorly understood. The spinal ejaculation generator (SEG), which expresses the neuropeptide, gastrin-releasing peptide (GRP), can trigger ejaculation in rats. Therefore, we focused on systemic effects of oxytocin on the GRP/SEG neuron system in the lumbar spinal cord controlling sexual activity in male rats. We found that systemic administration of oxytocin significantly shortened the latency to the first mount, intromission and ejaculation during male copulatory behavior. In addition, the local oxytocin level in the lumbar cord was significantly higher in males than in females. Histological analysis showed that oxytocin-binding is apparent in spinal GRP/SEG neurons. We therefore conclude that oxytocin influences male sexual activity *via* the SEG.

## Introduction

The neural network controlling male sexual activity consists of a number of components in both the brain and spinal cord [1,2]. In the rodent spinal cord, male sexual function is regulated by several specific centers, which are referred to as the spinal pacemaker[3], central pattern generator[4], spinal pattern generator [5,6], spinal ejaculation generator (SEG) [7,8]. SEG neurons trigger ejaculation at the spinal level, and they contain neuropeptides such as galanin [7–9], enkephalin[10], cholecystokinin [11,12], and gastrin-releasing peptide (GRP) [13] as neuromodulators. GRP neurons are important constituents of the SEG; they project to the somatic spinal nucleus of the lower lumbar-upper sacral spinal cord, which innervates the spinal autonomic nuclei and the bulbospongiosus muscles at the base of the penis [2,14,15]. We have previously reported that the spinal GRP neurons are greater in number and immunoreactivity in males than in females, showing a male-biased sexual dimorphism and appears to regulate at the spinal cord level male sexual functions such as erection and ejaculation in rodents (rats and mice) [13,16], non-rodent placental mammals (Eulipotyphla; Asian house musk shrews)[17], and primates (Japanese macaque monkeys)[18]. However, it remains unclear how the brain regulates these sexual centers in the cord.

While many people consider oxytocin to be a female reproductive hormone (oxytocin is derived from the Greek terms meaning “quick birth”) and it is often referred as the “love hormone”, it is reported that, in men, the plasma oxytocin level importantly increases after ejaculation [19,20]. However, this aspect of oxytocin physiology is poorly understood. We therefore focused, in this study, on systemic effects of oxytocin on the GRP/SEG neuron system controlling male sexual activity in rats.

## Results

**Figure 1.**
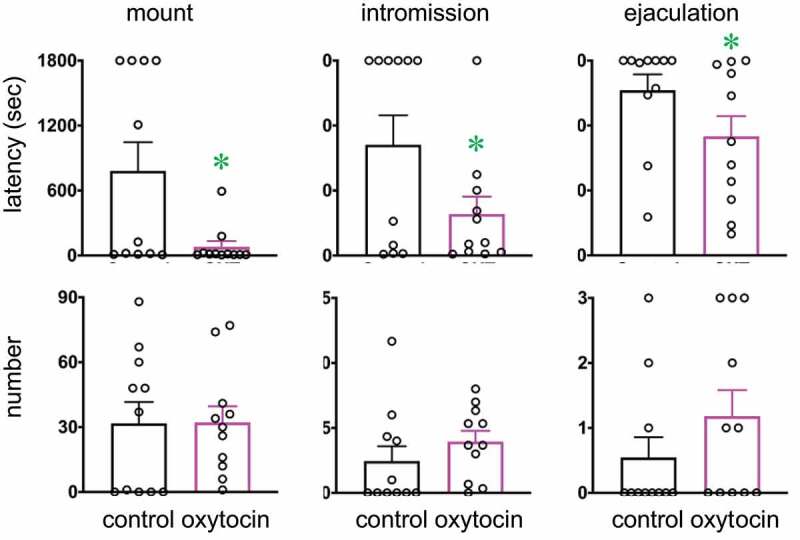
Effects of intraperitoneal administration of oxytocin on male sexual activity. Oxytocin shortens latencies to the first mount, intromission, and ejaculation [Data are presented as mean ± SEM and individual point (black), *n* = 11; paired *t* test, Mount latency: *t*_10_ = 2.53; Intromission latency: *t*_10_ = 2.30; Ejaculation latency: *t*_10_ = 2.55; Mount number: *t*_10_ = 0.03; Intromission number: *t*_10_ = 1.26; Ejaculation number: *t*_10_ = 1.88, *P < 0.05]

**Figure 2.**
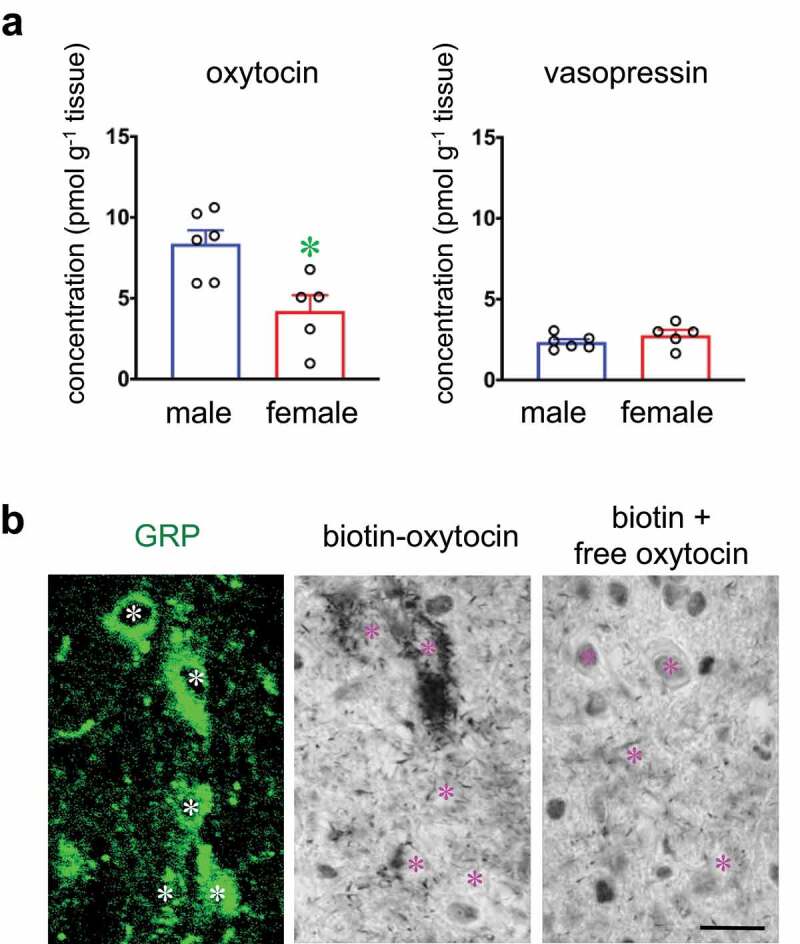
(a) Local concentrations of oxytocin and vasopressin in the lumbar spinal cord. [Data are presented as mean ± SEM (highlight) and individual point (black). Student’s unpaired *t* test; oxytocin, *t*_9_ = 3.25, *P < 0.05; vasopressin, *t*_9_ = 1.19, *P* = 0.133, male rats (*n* = 6), female rats (*n* = 5).] (b) Oxytocin-binding is apparent in the spinal GRP neurons (green). Left panel indicates the GRP^+^ neuronal cell bodies (green). Middle panel shows that oxytocin-binding is detected in the cytoplasm (chromogen aggregates) of GRP^+^ neuronal cell bodies. Right panel indicates the negative control sections incubated with biotinylated oxytocin and excess free oxytocin. *Asterisks* indicate the location of neuronal nuclei expressing GRP and oxytocin-binding double-positive neurons or binding-negative neurons (control). Scale bar: 20 µm

First, we examined the effects of systemic oxytocin treatment on male sexual activity (Figure 1). Systemic treatment of oxytocin significantly shortened the latency to the first mount, intromission, and ejaculation. In contrast, no significant change in numbers of mounts, intromissions, and ejaculations was observed. Subsequently, we measured the local concentration of oxytocin and vasopressin in the lumbar spinal cord (L2–L3; containing GRP/SEG neuronal cell bodies) (Figure 2a). The local concentration of oxytocin in the lumbar cord was significantly higher in males than in females, but that of vasopressin was relatively low, and did not differ between males and females (Figure 2a). Oxytocin-binding to spinal GRP neurons was examined histochemically (Figure 2b). Because GRP neurons are an important component of the SEG, we used GRP-immunocytochemistry to identify the SEG neurons. Incubation of sections of L2–L3 spinal cord with biotinylated oxytocin showed the accumulation of oxytocin (middle panel), especially in relation to the GRP-immunoreactive neurons in the lumbar cord (left panel, green). Control incubations with biotinylated oxytocin and excess free ligand showed the specificity of this binding (right panel).

## Discussion

In this study, we examined the effects of systemic treatment of oxytocin on sexual activity in male rats. In particular, we found that oxytocin systemic treatment significantly shortened the latency to the first mount, intromission and ejaculation, suggesting an increase in sexual activity. In men, it is suggested that an increased oxytocin level in the circulation influences libido, penile reflexes, and orgasm [21–23]. In male rats, oxytocin levels in cerebrospinal fluid doubled 5 minutes after ejaculation and tripled 20 minutes after ejaculation[24]. In contrast, plasma vasopressin in men increases during sexual arousal but it returns to baseline values by the time of ejaculation[19]. Vasopressin release during arousal might be associated with specific suppression of oxytocin until ejaculation[19]. In addition, intracerebroventricular administration of oxytocin to male rats has been reported to increase in the number of penile erections and yawning episodes in a dose-dependent manner [25–27]. We found in this study that systemic treatment of oxytocin promoted sexual activity, suggesting that oxytocin acts to control male sexual activity not only as a neuromodulator in the spinal cord but also as a circulating hormone.

We have reported recently that the distribution of oxytocin-neurophysin immunoreactive fibers is male-dominant in the lumbar spinal cord in rats[28]. In this study, local concentrations of oxytocin and vasopressin in the lumbar spinal cord (L2–L3; containing GRP/SEG neuronal cell bodies) were measured in rats, and oxytocin was significantly higher in males than in females, but AVP was relatively low with no sex difference. We have recently reported that GRP/SEG neurons in the lumbar cord express oxytocin receptors[28]. Indeed, our present results show the oxytocin binding in GRP/SEG neurons. We also demonstrated recently that oxytocin-containing axons projecting to the lumbar cord release oxytocin locally, and that oxytocin released there influences male sexual activity[28].

Magnocellular oxytocin-producing neurons in the paraventricular nucleus (PVN) and supraoptic nucleus of the hypothalamus release oxytocin into the circulation from the posterior pituitary to act systemically[29]. In contrast, the oxytocin-containing axons projecting into extrahypothalamic brain regions and the spinal cord are thought to be derived from parvocellular oxytocin neurons of PVN and to act locally at the projection sites [30–33]. It remains to be determined which of these two sources of oxytocin is more important in the action on the GRP/SEG system. We previously reported, in the lumbar cord, that the intrathecal administration of an oxytocin receptor antagonist significantly prolongs intromission latency and reduces the number of intromissions and ejaculations[28]. In addition, it has been suggested that oxytocin released in the lumbar cord may function as ‘switching’ between erection (parasympathetic nervous system) and ejaculation (sympathetic nervous system) [28,34]. Because systemic treatment with oxytocin significantly shortened the latency to the first mount, intromission, and ejaculation, we propose that oxytocin released into the circulation systematically facilitates male sexual activity, including sexual motivation, as a hormonal regulator. This is consistent with the finding of increased plasma oxytocin in the early pre-ejaculatory phases of sexual activity in naïve male rats[35]. Plasma oxytocin levels increased only after ejaculation in sexually experienced rats [35] as is reported for men [19,20]. Oxytocin released into the circulation therefore appears to influence sexual motivation, whereas oxytocin released locally in the lumbar cord exerts control sexual function at the spinal level. However, further studies are needed to determine precisely how the activity of magnocellular and/or parvocellular oxytocin neurons in the PVN can synchronize and release oxytocin both into the circulation and locally in the spinal cord during sexual behavior. Magnocellular oxytocin neurons in the supraoptic nucleus also release oxytocin into the circulation but perhaps not into the spinal cord directly. Whatever the outcome of these studies, activation of GRP/SEG neurons by oxytocin is clearly required for normal ejaculation.

We conclude that oxytocin facilitates male sexual activity at the spinal cord level *via* oxytocin receptors expressed on GRP/SEG neurons. While oxytocin is often thought to be primarily a ‘female’ reproductive hormone, it is now clear that oxytocin produced in hypothalamic neurons can **influence** male sexual activity both *via* the circulation and *via* direct efferents.

## Materials and methods

### Animals

Adult 11 Sprague-Dawley (SD) male rats (Shimizu Laboratory Supplies Co., Ltd., Kyoto, Japan or Charles River Japan, Yokohama, Japan) were used for sexual behavior tests. Adult 6 males and 5 females (SD strain; age, 2–4 months old) were used for ELISA. Adult 4 males (SD strain; age, 2–4 months old) were used for histochemical analysis. Two rats were group housed in polycarbonate cage (44 × 28 × 21 cm). All rats were maintained on a 12-h light/12-h dark cycle and were provided unlimited access to water and rodent chow. The Committee for Animal Research, Okayama University, Japan authorized the experimental procedures.

### Systemic administration of oxytocin

Oxytocin (AnaSpec Inc.) was intraperitoneally injected to sexually active males (250 nmol 500 µl^–1^ in PBS) 20-min (including a period for 5 min-adaptation in the test cage) before the behavioral tests, male rats were then subjected to sexual behavior tests (*n* = 11).

### Sexual behavior test

For sexual behavior tests, stimulus females were ovariectomized and estradiol benzoate (5 µg 0.1 ml^−1^ of sesame oil) was subcutaneously injected 3-d prior to testing. Progesterone (500 µg 0.1 ml^−1^ of sesame oil) was subcutaneously injected 4 − 6 h prior to testing to induce sexual receptivity. Sexual behavior tests were performed for 30 min and the latency of the first mount, intromission and ejaculation and the number of mounts, intromissions and ejaculations were counted.

### Peptide extraction and enzyme-linked immunosorbent assay (ELISA)

The concentrations of oxytocin and vasopressin in the upper (L3–L4 level) lumbar spinal cord were quantified by a competitive ELISA using a kit for oxytocin or vasopressin (Phoenix Pharmaceuticals, Burlingame, CA). Adult male (*n* = 6) and female (*n* = 5) rats were used in this study. All rats were killed by decapitation under deep sodium pentobarbital anesthesia between 10:00 and 12:00 h. Lumbar spinal cords (L3–L4 level) were quickly removed on ice, weighed, snap-frozen immediately in liquid nitrogen, and used for peptide extraction. Peptides were extracted according to our previous methods[36]. In brief, frozen tissues were homogenized in 5% acetic acid using BioMasher (Nippi, Tokyo, Japan) and boiled for 7 min. The homogenate was centrifuged at 15,000 x *g* for 10 min at 4°C. The supernatant was collected in a tube, and the precipitate was again homogenized and centrifuged. The two supernatants were pooled and forced through a disposable C-18 cartridge (SPE, 1 ml-100 mg; SILICYCLE, Quebec, Canada). The retained material was then eluted with 60% methanol. The elute was concentrated in a vacuum centrifugal concentrator (CC-100, TOMY SEIKO, Tokyo, Japan) and subjected to competitive ELISA for oxytocin or vasopressin using a kit (Phoenix Pharmaceuticals, Burlingame, CA) according to the manufacturer’s protocol. The concentration of oxytocin and vasopressin was calculated in terms of picomoles per gram wet weight (pmol g^–1^ tissue) of each spinal cord. In terms of ELISA for oxytocin: the inter- and intra-assay variations were less than 15 and 10%, respectively. The minimum detection limit was 0.09 ng ml^–1^. In terms of ELISA for vasopressin: the inter- and intra-assay variations were less than 14 and 7%, respectively. The minimum detection limit was 0.04 ng ml^–1^.

### Oxytocin binding

Binding assay was performed as described previously[13]. After perfusion with zinc-buffered formalin (10% v/v), spinal cords (*n* = 4 male rats) were post-fixed overnight in the same fresh fixative solution and then transferred to 70% ethanol prior to processing through paraffin embedding. Five-micron sections were prepared with a microtome (Leica, Nussloch, Germany) and placed on positive charged slides. The slides were then deparaffinized in xylene and rehydrated through graded alcohols to water, and processed first for GRP-immunofluorescence with Alexa Fluor 488-linked anti-rabbit IgG (Molecular Probes, Eugene, OR) as described previously [13,28]. The stained sections were then photographed under a confocal laser scanning microscopy. Subsequently, oxytocin binding assay was performed as described previously [13,28]. Antigen retrieval was performed by immersing the slides in Target Retrieval Solution (Dako Corp., Carpinteria, CA) for 20 min at 98°C. After cooling at room temperature for 30 min, the sections were washed in water. All incubations were done at room temperature, and Tris-buffered saline containing 0.05% Tween 20 (pH 7.4) (TBST) was used for all washes and diluents. Slides were blocked with 1% BSA in TBST for 30 min and after washing, 1 µg ml^–1^ biotinylated rat oxytocin (AnaSpec, Inc., San Jose, CA) was added to the slides and incubated for 2 h at room temperature. Oxytocin binding was detected with a streptavidin-biotin kit (Nichirei, Tokyo, Japan), and then the tetramethylbenzidine reaction followed by diaminobenzidine-nickel method was used to visualize the bound SAB-horse radish peroxidase-oxytocin complexes as described previously [18,28,37]. Stained sections were viewed using an Olympus Optical (Tokyo, Japan) BH-2 microscope. The unlabeled oxytocin (1 µg ml^–1^) was used as a negative control, in which case no specific labeling was detected (data not shown). Binding competitions with biotinylated oxytocin were also conducted by using the excess amounts (100 µg ml^–1^) of unlabeled rat oxytocin. Binding studies were repeated four times and produced similar results using independently fixed spinal cords from different animals.

### Statistics

Statistical analyses were performed using GraphPad Prism 8 (8.4.3; GraphPad Software, San Diego, CA). Data are presented as mean ± standard error of the mean (SEM) and individual point (black).
